# Coronal alignment in total knee arthroplasty: a review

**DOI:** 10.1186/s10195-023-00702-w

**Published:** 2023-05-22

**Authors:** F. Matassi, F. Pettinari, F. Frasconà, M. Innocenti, R. Civinini

**Affiliations:** Orthopedic Clinic, AOU Careggi, University of Florence, Florence, Italy

**Keywords:** Total knee arthroplasty, TKA, Coronal alignment, Knee alignment, Robotic surgery, Personalized alignment

## Abstract

Total knee arthroplasty (TKA) alignment has recently become a hot topic in the orthopedics arthroplasty literature. Coronal plane alignment especially has gained increasing attention since it is considered a cornerstone for improved clinical outcomes. Various alignment techniques have been described, but none proved to be optimal and there is a lack of general consensus on which alignment provides best results. The aim of this narrative review is to describe the different types of coronal alignments in TKA, correctly defining the main principles and terms.

## Introduction

Coronal alignment in total knee arthroplasty (TKA) has gained increasing attention since considered a cornerstone to improve clinical outcomes. To overcome the problem of patient dissatisfaction and perception of “unnatural knee” after TKA, different alignment options and philosophies have been described with the purpose to better reproduce knee anatomy and kinematics.

**Fig. 1:**
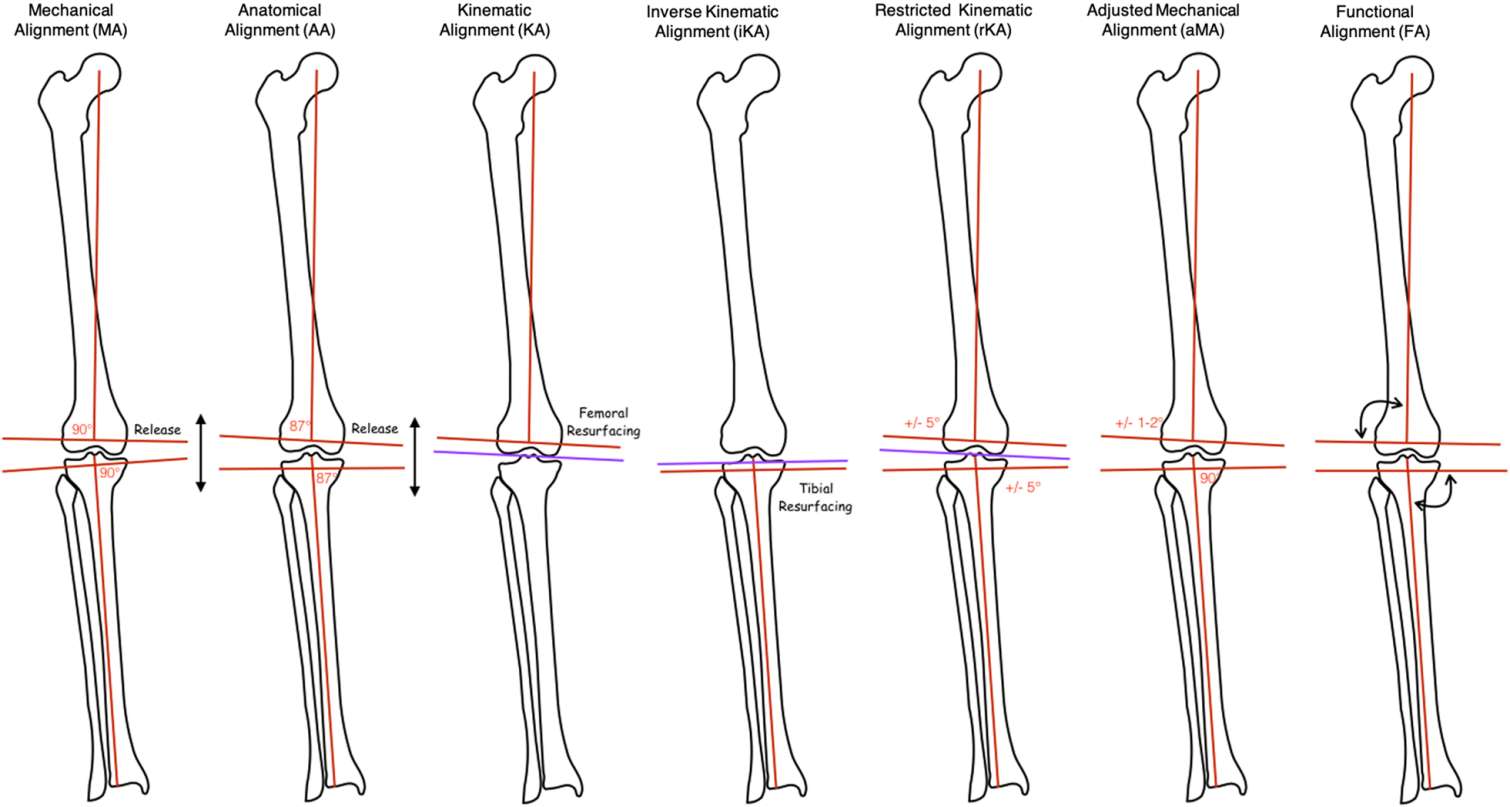
Various alignment techniques

Nowadays different principles and surgical techniques have been described that can be classified in three main categories [1] (Fig.1, Table 1):

1. *Systematic alignment*, which includes mechanical alignment (MA) [2–5] and anatomic alignment (AA) [7] with the goals to restore neutral alignment with hip–knee–ankle axis (HKA) of 180° for all patients independently from preoperative alignment;
2. *Patient-specific alignment* such as kinematic alignment (KA) [13] that aims to maintain the native limb alignment and joint line inclination;
3. *Hybrid alignment* such as restricted kinematic alignment (rKA) [24, 25], inverse kinematic alignment (iKA) [23–25], adjusted mechanical alignment (aMA) [28–32], and functional alignment (FA) [35, 36] with the aim to restore the coronal alignment within an HKA angle safe zone of 177° to 183°.

**Table 1:** Main key points in different coronal alignments philosophies

	Mechanical alignment (MA)	Anatomic alignment (AA)	Kinematic alignment (KA)	Inverse kinematic alignment (iKA)	Restricted kinematic alignment (iKA)	Adjusted mechanical alignment (aMA)	Functional alignment (FA)
Distal femoral cuts	90°	93°	Femoral resurfacing	According to extension gap	90 ± 5°	90 ± 2°	According to extension gap(± 3°)
Proximal tibial cuts	90°	87°	According to extension gap	Tibial resurfacing restricted to 84° (varus) to 92° (valgus)	90 ± 5°	90°	According to extension gap (± 3°)
Femur external rotation to PCA	3°	0°	Femoral resurfacing	According to flexion gap	According to flexion gap	3°	According to flexion gap
Overall alignment (HKA)	0°	0°	Native alignment	Slight undercorrection safe zone +6° varus to −3° valgus	Slight undercorrection safe zone +6° varus to −3° valgus	Slight undercorrection	Slight undercorrection
Ligament release	Yes	Yes	No	Minimal	Minimal	Minimal	Minimal
Type	Systematic	Systematic	Patient specific	Hybrid	Hybrid	Hybrid	Hybrid

To date there is no consensus on the optimal coronal alignment techniques, and further studies with larger samples and longer follow-ups are necessary to prove which technique has more benefits than others. However, beyond clinical studies what is unclear is a correct definition of terms in the plethora of names used for defining each type of alignment.

The aim of this narrative review is to clarify the different types of coronal alignment in TKA with correct definition of the main principles. We believe this narrative will help readers and researchers have a more universal definition of terms facilitating comparable analysis and clinical studies.

### Mechanical alignment (MA)

#### Principles

Mechanical alignment in TKA was described by Ranawat and Insall in the 1970s and is the most commonly used in TKA with well-documented long-term results. The principle of this type of alignment is to position both the femoral and tibial components perpendicular to the mechanical axis. This allows one to obtain, after proper ligament release, a hip–knee–ankle (HKA) angle of 180°. Neutral alignment guarantees symmetric balanced load distribution between the medial and lateral compartments that minimize wear and potential component loosening. This alignment introduced the “compromise of 3°” as the femoral component should be positioned with 3° of external rotation to balance flexion gaps with the extension gaps [2].

#### Clinical results

The mechanical alignment has been considered the gold standard for decades, and many studies have reported satisfactory clinical outcomes and long-term survival of implants between 89% and 99% at 10 years and between 85% and 97% at 20 years of follow-up [2–4]. Clinical results [Oxford Knee Score (OKS), Western Ontario and McMaster University index (WOMAC), Knee Society Score (KSS), range of motion (ROM)] were considered excellent with improvement in all outcomes from preoperative to postoperative [5].

#### Criticisms

However, recently many criticisms have been raised regarding this principle, with some studies showing that up to 20% of patients were dissatisfied after TKA. One of the reasons could be attributed to the fact that MA is a systematic alignment where all limbs are aligned to a neutral HKA axis independently of the preoperative alignment. There is a wide individual variation in limb alignment, and the neutral condition can be “unnatural” in most patients. Bellemans et al. showed that more than 30% of male non-arthritic patients had a constitutional varus angle of > 3° [28]. Also, Hirschmann et al. showed a wide distribution of femoral and tibial coronal alignment in young non-osteoarthritic knees [6]. For this reason, positioning the tibial and femoral component perpendicular to the mechanical axis will lead to a situation different from the native knee with abnormal joint line obliquity and alteration of normal knee biomechanics that could negatively influence clinical results in TKA.

### Anatomic alignment (AA)

#### Principles

During the 1880s Hungerford and Krackow first described the anatomic alignment concept to reproduce the “anatomic” oblique joint line after total knee replacement [7]. The aim of AA is to position the femoral component in a fixed position of 3° of valgus and the tibial component in 3° of varus relative to the mechanical axis of the limb. In this way it is possible to restore the native knee anatomy and the natural joint line inclination of 3° preserving periarticular knee tension during the full range of motion. In this technique the overall limb alignment, after proper ligament release, is restored to neutral (HKA 180°) in all patients, and for this reason it is also considered a systematic approach. Since the femoral and tibial components are placed in 3° of inclination, the need to externally rotate the femoral component to balance the flexion gap is obviated and the femoral component is aligned parallel to the posterior condylar axis (PCA).

#### Clinical results

This concept is supported by some clinical and cadaveric studies that found a more stable implant during the full range of motion with less ligament release required during the procedure with AA compared with MA. Preliminary studies have reported good clinical outcomes but with short-term follow-up. However, there is a lack of long-term data on implant survival that support the varus alignment of the tibial component [8, 9].

#### Criticisms

The concept was criticized on the basis of the technical difficulties in performing the varus cut on the tibia in a precise and reproducible way. The main concern with AA is that inadvertent over-resection of more than 3° in the proximal tibial cut may lead to excessive varus of the tibial implant, which is associated with premature component failure in TKA [10, 11]. Moreover, another important drawback with this technique is the use of first-generation tibial keel design that provided poor tibial fixation compared with the new-generation implants [12]. The results of this type of alignment should be analyzed in the light of the new prosthetic designs available. Due to the absence of reproducibility in the surgical technique and long-term results on implant survival, this type of alignment was progressively abandoned.

### Kinematic alignment (KA)

#### Principles

Introduced by Howell et al. in 2008, this technique aims to restore the pre-arthritic hip–knee–ankle angle, the pre-arthritic joint line obliquity, and thus the natural tension of the ligaments.

The KA is a “true femoral resurfacing” where the femoral joint line level is restored by removing cartilage and bone thickness equivalent to the implant thickness [13].

This technique is occasionally called “calipered” technique since the use of caliper is essential for measuring the desired resection. The flexion and extension gaps are balanced consequently with the tibial resection.

Restoring the natural joint line, the femoral component is aligned parallel or perpendicular to the three main knee kinematic axes (transcondylar or cylindrical axis; patellar axis; tibial longitudinal axis), which dictate the physiological knee motion. In fact, similar to unicompartmental knee replacement, KA TKA restores the constitutional joint line on the femoral side and the physiological knee laxity without the need for soft-tissue release. For this reason, it is also considered a patient-specific technique and a procedure involving only bone.

#### Clinical results

There are many studies that compare KA and MA alignment techniques, but the results are quite unpredictable and in contrast to each other. Some studies have shown improved clinical outcomes in KA compared with MA at short-term follow-up, while others have shown no difference in clinical or functional outcomes between the two alignment techniques [14–17].

Young et al. in a randomized controlled trial of 99 TKAs found no difference in functional outcomes, survivorship, or radiographic signs of aseptic loosening between KA and MA at 5 years of follow-up [18]. Courtney and Lee pooled the data of 877 KA TKAs from nine studies and reported a survival rate of 97.4% at 38 months of follow-up [20].

However, there are some limitations within existing studies about functional outcomes, component survivorship, and long-term complications. In fact, studies are very heterogeneous in the choice of the preoperative planning method, intraoperative alignment technique using jigs, 3D cutting blocks, patient-specific implants, or computer-assisted procedures. Studies with longer follow-ups and standardized techniques with larger cohorts are required to prove any benefit of the KA over MA technique.

#### Criticism

One of the main concerns regarding KA is the varus or valgus outlier range of the tibial component and limb alignment that might adversely affect the long-term results. Some biomechanical studies showed that the varus position of the tibial component is associated with increased polyethylene wear, risk of varus collapse due to bone stress, and altered ligament strains as compared with the neutral aligned model [22]. However, in a retrospective review of 222 primary KA TKAs, Howell et al. showed that the aseptic revision rate at 10 years follow-up was 1.6%, with implant survivorship of 97.5%. The varus tibial component did not adversely affect the 10 year implant survival, yearly revision rate, and level of function [19].

Another critical point is the potential risk of patellofemoral instability due to the lack of external rotation of femoral component. However, some data showed that there was no increase in the rate of patellofemoral complications in the KA group compared with the MA group [18]. In a meta-analysis of 229 KA and 229 MA knees comparing the revision rate for patellofemoral complications, there was no difference between the two groups (1.3% versus 1.3%) [20]. Another study reported only 13 cases of patellar instability out of 3212 KA TKAs [21]. However, there are sufficient data in the literature to state not only that there is no increase in the rate of patellofemoral complications [18] but also that the KA shows a better overall restoration of patellar kinematics compared with a conventional mechanical alignment technique [22].

Another concern is related to the use of nonspecific implants. Since the goal of KA is to reconstruct the individual pre-arthritic limb alignment giving a more natural feeling of the knee to the patient, the use of more anatomic or patient-specific femoral components instead of standard implants designed and biomechanically tested for perpendicular stresses is reasonable [13].

### Inverse kinematic alignment (iKA)

#### Principles

Recently, Winnock de Grave described the inverse kinematic alignment (iKA), which aims to resurface the proximal tibia (“true tibial resurfacing”) with equal medial and lateral resections maintaining the native tibial joint line obliquity [24]. If some amount of bony wear/bone loss is present on the medial tibial plateau, its estimation should be considered and removed from the total amount of bone resection planned for the opposite lateral tibial plateau to avoid an excessive varus tibial resection and an extreme joint line obliquity. The flexion and extension gaps are therefore balanced by adjusting the femoral resections with no soft tissue or minimal releases [25, 26]. The technique was first described with the use of robotic surgery to achieve more accurate resection to restore the pre-arthritic medial proximal tibial angle (MPTA), remaining in a “restricted” safe zone of 84° (varus) to 92° (valgus). The tibial slope was also set equal to the native medial tibial slope. This technique is considered a hybrid alignment as it aims to maintain the native coronal alignment within a HKA angle safe zone of 174° to 183°.

It can be considered an evolution of KA with the advantages of reducing the varus position of the tibial component; in fact, in some cases, KA involves complex algorithms to balance the flexion and extension gaps, which may result in more oblique tibial varus resections with high risk of failure. The iKA results in a less varus postoperative tibial joint line of 3 ± 2° due to the tibial reference compared with the femoral reference in KA.

#### Clinical studies

There are only few studies that analyze the clinical outcomes using this technique with no long-term data available on survival rate since it has been recently described.

Winnock de Grave et al. reported no significant difference in clinical results at 12 months between iKA and aMA. They found a higher rate of satisfaction and significant improvement in postoperative OKS for restricted inverse KA [24].

#### Criticism

Only early results are available, and further studies are needed to validate the clinical benefits and long-term outcomes of iKA compared with the other types of alignment.

### Restricted kinematic alignment (rKA)

#### Principles

In 2011 Vendittoli proposed the restricted kinematic alignment (rKA) protocol, setting boundaries to KA for patients with an outlier or atypical knee anatomy, to avoid excessive coronal deviation [25]. The rKA follows the main technical principle of KA technique, respecting as much as possible the natural alignment of the femoral component within certain limits of "restriction.”

The first pillar of this protocol is to reproduce individual lower limb anatomy while keeping a HKA within ± 3°. The second pillar is to reproduce the individual’s anatomy keeping LDFA and MPTA within ± 5°. Applying these rKA principles, 51% of the population would undergo a classic KA without any modification, another 30% would have a correction of < 1°, and the remaining 20% of patients would require more substantial adjustments and slight ligament releases [26].

#### Clinical results

A clinical series of 100 cemented rKA TKAs demonstrated satisfactory functional outcomes at 2.4 years follow-up. Minor ligamentous releases were required in only 5% of the knees [27]. Another study presented 100 cementless rKA TKAs without any revision for aseptic loosening at 49 months of follow-up [28]. Blakeney et al. found that postoperative gait patterns of rKA TKAs were significantly closer to healthy controls compared with MA [27].

#### Criticism

Currently, no study has assessed the mid- or long-term clinical outcomes after rKA TKA.

### Adjusted mechanical alignment (aMA)

#### Principles

aMA is an adaptation of classical MA with the aim to restore the preoperative constitutional deformities with TKA. Implant position adjustment are made on femoral side while the tibial component is placed perpendicular to mechanical axis according to MA principles [29, 30].

The rationale of this alignment is to preserve the constitutional frontal plane deformity with an accepted range up to 6° of residual varus or valgus deformity thus reducing the needed for ligament release to balance the flexion–extension gaps.

This is considered a “hybrid technique” as the tibial component is systematically positioned at 90° and the femoral component is personalized according to the patient’s anatomy [31–33].

#### Clinical results

Vanlommel et al. in a cohort of 143 varus knees reported better clinical results and functional outcomes after TKA if the postoperative alignment was left in mild residual varus compared with the neutral aligned knees, underling the importance of under-correction of the preoperative deformities. Comparing the MA versus aMA technique, no differences were reported at 1 year follow-up even if there was higher HSS score at 1 month and 6 months postoperatively for aMA TKAs [31]. Winnock de Grave et al. [32] reported a consecutive series of 80 patients that received robotic-assisted TKA with restricted iKA (*n* = 40) and with aMA (*n* = 40), and they found no differences in clinical outcomes at 12 months of follow-up.

#### Criticisms

Actually, no study demonstrates the superiority of aMA technique in terms of clinical outcomes and long-term survival of the implants rather than classic MA [34, 35].

Severe knee deformity (> 20° varus) could be treated with this technique, but this requires an important medial ligamentous release for balancing the flexion–extension gap.

To restore the physiologic obliquity of a patient’s joint line, a specific implant is also necessary.

### Functional alignment (FA)

#### Principles

FA is considered an evolution of the KA method. The aim of this technique is to restore the natural obliquity of joint line and balance the knee flexion–extension gap by fine-tune adjustments of tibial and femoral components, avoiding soft tissue releases [36].

The use of robotic technology is a prerequisite to assess implant position, resection thickness, joint gaps, and limb alignment during surgery. The first step is to use a computed tomography (CT) scan of the knee for creating a realistic reconstruction of patient’s knee with which the surgeon can plan bone cuts obtaining the desired positioning of the implants and limb alignment. The femoral component in the coronal plane is inclined from a starting point of 0° to the mechanical axis to achieve the correct balancing between medial and lateral compartment. In the sagittal plane, the component is positioned to avoid femoral notching and to follow the natural bone’s bowing. In the axial plane the implant is aligned starting to the transepicondylar axis and moving ± 3° to balance the flexion gap. On the other side, the tibial component is positioned to restore natural joint line inclination in coronal and sagittal plane avoiding valgus position [37]. Minimal adjustment on the tibial position can be done to balance the knee. Soft tissue release must be considered only in case of severe fixed deformity that impedes the gap balancing with bone cuts.

#### Clinical results

Kayani, the main promoter of this alignment, reported comparable WOMAC score in MA TKAs versus FA TKAs 2 years after surgery in 100 patients [36]. Recently, Clark et al. in their prospective cohort of 300 knees undergoing cruciate retaining total knee arthroplasty found that functional alignment more consistently achieves a balanced total knee arthroplasty than either mechanical or kinematic alignment prior to undertaking soft tissue release [38].

In a single-center retrospective cohort study of 110 consecutive TKAs performed with an image-based robotic system, the ligament balancing in the medial and lateral femorotibial compartments was assessed intraoperatively. The authors found that KA failed to deliver a balanced TKA in more than 50% of cases, especially regarding the flexion gap compared with FA [39].

#### Criticisms

No study investigated the functional and clinical outcomes of this alignment technique or the implant survivorship. Furthermore, the use of robotic technology presupposes a rise of costs for additional preoperative imaging and increasing operation times during learning curve [40].

## Conclusion

Coronal alignment is considered a cornerstone to address the unresolved problem of patient dissatisfaction and the perception of an unnatural knee after TKA. Nevertheless, to date, there is no consensus on the optimal coronal alignment. The scientific literature is confusing and lacks a clear definition of terms in the plethora of different types of alignment.

With the purpose of establishing a clear definition and a universal language for the scientific community, this narrative review summarizes each type of alignment with the underlying main principles and definitions.

TKA is a complex procedure, and the coronal plane represents only one of the three-dimensional planes that could influence clinical outcomes. Sagittal and rotation alignment largely affect the postoperative kinematics and clinical results, and their importance cannot be neglected. Abnormal internal or external rotational alignment of the tibial or femoral component leads to patellofemoral maltracking, and abnormal ligament tension during knee flexion can cause unexplained painful TKA. Achieving correct femoral and tibial rotation is difficult, with large variability among surgeons. Many landmarks are described with no evidence to date regarding the best methods. This topic, still unresolved, can be considered another cornerstone for a successful TKA [41]. Similarly, sagittal alignment of both femoral and tibial components can influence the flexion space, the postoperative range of motion, and thus patients’ satisfaction [42].

Another important drawback of the current literature is the absence of a standard surgical technique for the same type of alignment, which makes clinical results not comparable to each other. In most of the clinical research, the surgical technique is an adaptation of the conventional technique using mechanical jigs. Studies have shown that using standard jigs-based techniques for alignment falls outside ± 3° of the target in up to 30% of patients [43]. For this reason, the surgical technique could be considered an important bias that may influence results within the same type of alignment. A more precise and universal surgical technique is mandatory. In this sense, robotic surgery can help standardize the surgical technique and make bony resections and component alignment more reproducible to improve the comparability of clinical studies [37].

Furthermore, the components available nowadays are designed to be implanted with the MA technique. Since the goal of the new alignment philosophies is to reconstruct the patient’s specific limb alignment giving a more natural feeling of the knee to the patient, it is reasonable to use implants designed to better replicate the constitutional pre-arthritic knee anatomy [44].

Determining the optimal coronal alignment for patients undergoing TKA is one of the great challenges in reconstructive knee surgery.

Hirschmann et al. in 2019 introduced the concept that different functional knee phenotypes require an individualized approach to TKA coronal alignment. They introduced a novel classification with the purpose to individuate different alignment targets in patients with different non-osteoarthritic native alignments [6]. In 2021 MacDessi et al. published their coronal plane alignment of the knee (CPAK) classification that could help surgeons to determine which alignment strategy is best suited for each patient [45]. Despite all the terminologies, we still lack evidence on how much the preoperative alignment could affect the postoperative outcomes if addressed by different types of coronal alignment philosophies in patients presenting with varus deformities more than 20° as well as in patients with any kind of valgus deformity greater than 3°. For such cases, the literature is still almost all about the MA showing that postoperative neutral alignment results in longer TKA survival time than residual varus alignment [46].

High-quality studies with standard techniques are necessary to understand which type of alignment can be associated with better clinical outcomes, taking into consideration that many factors other than coronal alignment could not be neglected.

## Data Availability

Not applicable.

## Abbreviations

AA: Anatomical alignment
aMA: Adjusted mechanical alignment
FA: Functional alignment
HKA: Hip–knee–ankle axis
iKA: Inverse kinematic alignment
KA: Kinematic alignment
KSS: Knee Society Score
LDFA: Lateral distal femoral angle
MA: Mechanical alignment
MPTA: Medial proximal tibial angle
OKS: Oxford Knee Score
PCA: Posterior condylar axis
rKA: Restricted kinematic alignment
ROM: Range of motion
TKA: Total knee arthroplasty
WOMAC: Western Ontario and McMaster University index
